# A Second Look at the Use of VersaWrap® Nerve Protector in Spine Surgery: A Case Report

**DOI:** 10.7759/cureus.84686

**Published:** 2025-05-23

**Authors:** Adam Bruggeman, Kelly Van Schouwen, Robin Keswani

**Affiliations:** 1 Orthopaedic Surgery, Texas Spine Care, San Antonio, USA; 2 Orthopaedic Surgery, Research Source, Austin, USA

**Keywords:** nerve root tethering, orthopedic spine surgery, post-laminectomy syndrome, spine revision surgery, versawrap nerve protector

## Abstract

The presence of severe epidural fibrosis (EF) during a spinal revision procedure increases the risk of interoperative complications and post-operative success. We present a retrospective series of one surgeon’s standard of care application of VersaWrap^®^ Nerve Protector (Alafair Biosciences, Inc., Austin, TX, USA) in 169 lumbar decompressions in conjunction with Depo-Medrol (80 mg) and Marcaine/epinephrine (1 mL) to manage scar tissue tethering of the nerve root to the surrounding tissues. Of the 169 total lumbar decompression procedures, there were zero (0) complications reported for VersaWrap in conjunction with the medicinal agents in the dosages reported. A representative case of a patient who later underwent a revision procedure is highlighted to allow a second-look assessment of the initial VersaWrap application. The surgeon's experience was that VersaWrap bioresorbed completely and that any scar tissue present was easy to remove without significant mechanical disruption. We also discuss standard of care surgical practice with VersaWrap in conjunction with medicinal agents and future directives of VersaWrap application.

## Introduction

Epidural fibrosis (EF), the formation of scar tissue in the epidural space of the spine, following a spine surgical procedure is a well-documented contributor of failed back surgery syndrome (FBSS) and other complications including the need for reoperation [1]. FBSS is characterized by persistent or recurring pain in the back or legs after a spine procedure and the occurrence of FBSS varies among studies, with estimates from 8% to 20% [2,3]. One study found that EF caused the need for a revision procedure in 4% to 9% of postdisectomy cases [4]. The presence of postoperative EF significantly increases the technical difficulty and risk of complications during revision spinal procedures [5] and has been reported as the third most common cause of FBSS [1].

The use of perioperative adhesion barriers (or nerve protectors) to reduce the risk of complications in the presence of scar tissue is common. These adhesion barriers (or nerve protectors) have historically included carboxymethylcellulose/polyethylene oxide (CMC/PEO) [6], gelatin USP products [7], bacterial cellulose with mesenchymal stem cells [8], and carbohydrate polymer gels [9,10].

The formation of scar tissue begins with an inflammatory reaction in the first few days after a surgical procedure [11]. Current literature supports the prophylactic perioperative use of Depo-Medrol for immediate postoperative inflammation control [12] and Marcaine/epinephrine for pain management [13]. Adhesion barriers (or nerve protectors) used in conjunction with medicinal agents may offer promising patient outcomes and there is currently no standard of care for management of nerve root in conjunction with medicinal agents during spine procedures.

VersaWrap® Nerve Protector (Alafair Biosciences, Inc., Austin, TX, USA), is a thin, gelatinous hydrogel peripheral nerve protector that creates a protective layer between the nerve root and surrounding tissues [14]. VersaWrap is comprised of hydrophilic, biocompatible, bioresorbable biopolymers hyaluronic acid (non-animal) and alginate [15]. VersaWrap bioresorbs completely, does not remodel into additional tissue, and is designed to bioresorb over a period of three to six months [14]. The hydrogel is highly conformable and tissue-adherent, precluding the need for suturing or tissue glue [14]. VersaWrap’s conformability and bioresorbable nature may facilitate its integration with other intraoperative agents.

In this paper, we present a retrospective series of one orthopedic spine surgeon’s standard of care application method of VersaWrap Nerve Protector in conjunction with Depo-Medrol (80 mg) and Marcaine/epinephrine (1 mL) in 169 lumbar decompressions, from May 2018 to March 2020. A representative case of a patient who later underwent a revision procedure is highlighted to allow a second-look assessment of the initial VersaWrap application.

## Case presentation

Initial procedure: left L4-5 laminectomy

A 42-year-old male presented with chronic low back pain that had been ongoing for many years. He had a surgical history of an L5-S1 fusion and reported reinjury to his back. He had no other relevant medical or personal history. He had undergone multimodal conservative treatment through a series of epidural injections, physical therapy, and daily pain management with anti-inflammatory and narcotic medication use. He was diagnosed with spinal stenosis and elected for a left L4-5 laminectomy.

**Figure 1:**
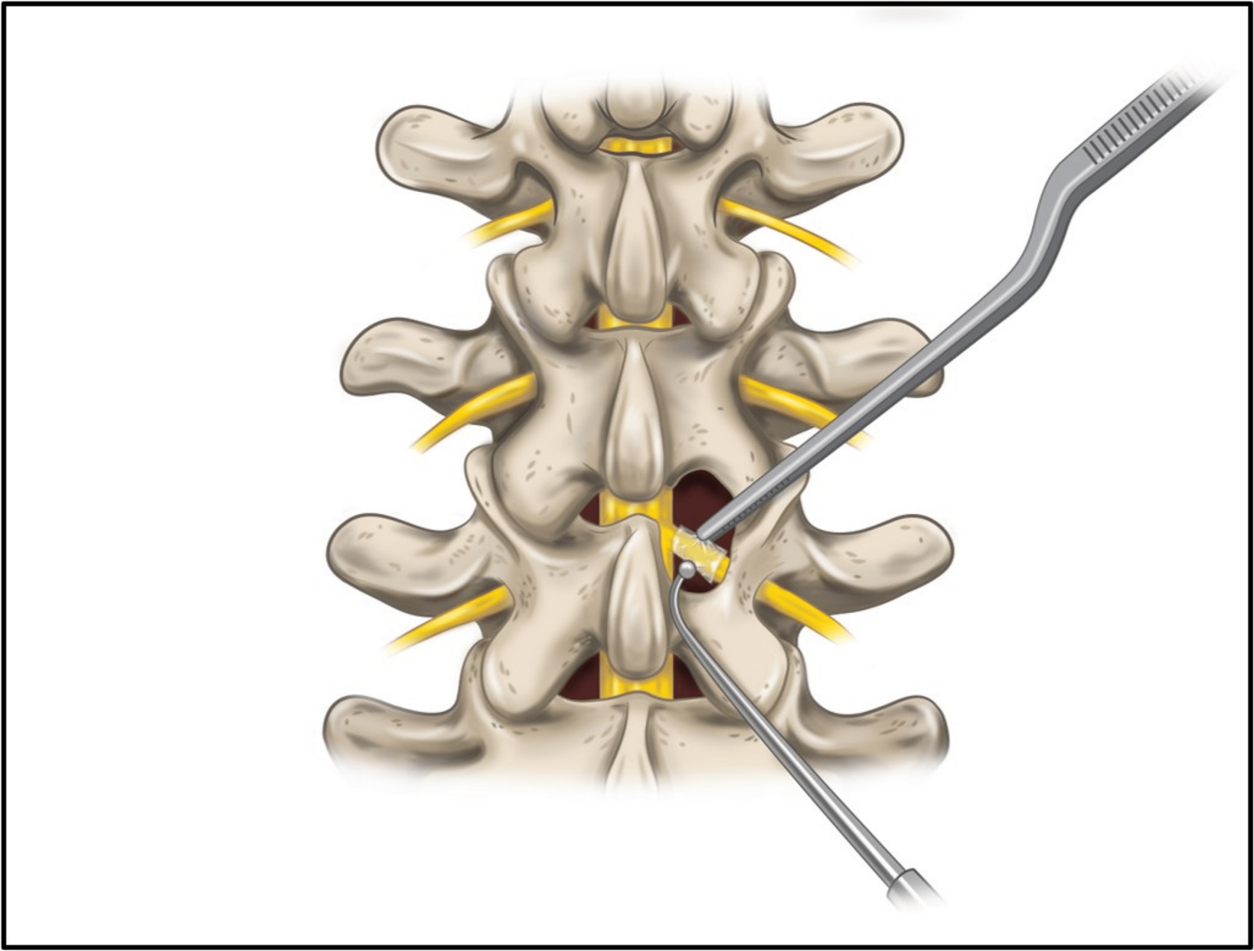
Top view illustration of VersaWrap Nerve Protector 1 cm x 1 cm square sheet application onto the peripheral nerve root and the adjacent dura, under the lamina of the spine in the laminectomy procedure. Image reproduced with permission from Alafair Biosciences, Inc., Austin, TX, USA

**Figure 2:**
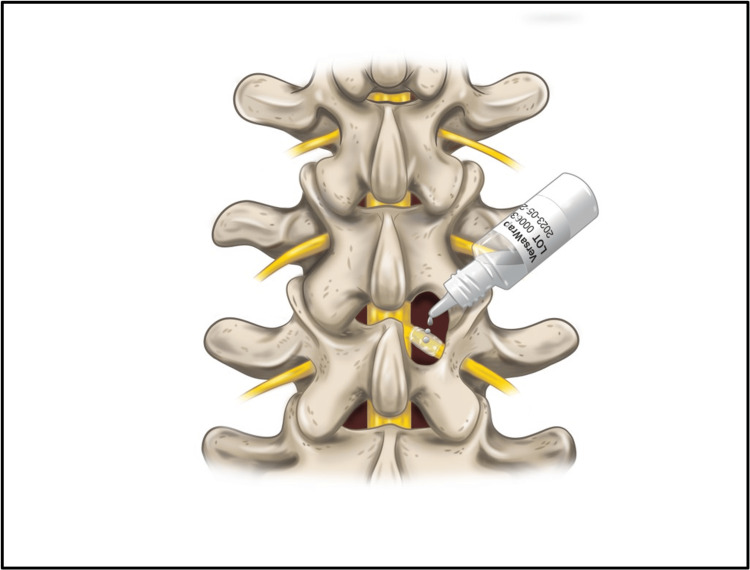
Top view illustration of VersaWrap Nerve Protector wetting solution dropwise application on implanted VersaWrap sheet on the peripheral nerve root and the adjacent dura, under the lamina of the spine in the laminectomy procedure to create a tissue-adherent, conformable, gelatinous hydrogel layer. Image reproduced with permission from Alafair Biosciences, Inc., Austin, TX, USA

Intraoperatively, the laminectomy was performed using the minimally invasive tubular retraction (MITR) technique. The VersaWrap sheet was cut into 1 cm x 1 cm squares, advanced through the tube, and placed onto the peripheral nerve root and the adjacent dura, under the lamina of the spine (Figure 1). The VersaWrap wetting solution was added dropwise to the sheet to create a tissue-adherent, conformable, gelatinous hydrogel layer (Figure 2). Depo-Medrol (80 mg) and Marcaine/epinephrine (1 mL) were applied separately on top of the VersaWrap to reduce immediate postoperative pain and inflammation.

Second procedure: left L4-5 revision laminectomy

At 21 months post-operative, the patient had recurrent disk herniation and significant spinal stenosis along with correlating radicular pain down his left leg. The patient was diagnosed with post-laminectomy syndrome and elected for a left L4-5 laminectomy revision.

During reoperation, the revision procedure was performed using the MITR technique. The surgeon noted that the previously applied VersaWrap (left L4-5 laminectomy) had completely bioresorbed. When he encountered scar tissue, he was able to easily remove this tissue from the nerve root and adjacent tissues without significant mechanical disruption.

Surgeon experience was that the scar on tissues where VersaWrap was not previously placed (L5-S1 fusion) was significantly thicker when compared to the scar on tissues where VersaWrap was applied in the initial laminectomy (Video 1).

**Video 1:** Video of scar on tissues being easily removed where VersaWrap Nerve Protector was applied in the initial left L4-5 laminectomy (left side). Thick scar on tissues where VersaWrap was not previously placed in the L5-S1 fusion (right side).

After the scar tissue was completely removed, the VersaWrap sheet was cut into 1 cm x 1 cm squares, advanced through the tube, and placed on the peripheral nerve root and the adjacent dura, under the lamina of the spine. The VersaWrap wetting solution was added to the sheet dropwise to create a hydrogel layer (Video 2). Depo-Medrol (80 mg) and Marcaine/epinephrine (1 mL) were applied separately on top of the VersaWrap to reduce immediate postoperative pain and inflammation.

**Video 2:** Video of VersaWrap Nerve Protector application during the revision laminectomy. The VersaWrap sheet was applied onto nerve root and then the VersaWrap wetting solution was applied onto the implanted VersaWrap sheet. Conformance to underlying tissues is observed with application of VersaWrap solution.

## Discussion

VersaWrap Nerve Protector was implanted as standard of care during 169 lumbar decompressions in conjunction with Depo-Medrol (80 mg) and Marcaine/epinephrine (1 mL) to manage scar tissue tethering of the nerve root to the surrounding tissues such as bone, dura, and muscle. Of the 169 total lumbar decompression procedures, there were zero (0) complications reported for VersaWrap application in conjunction with Depo-Medrol and Marcaine/epinephrine in the dosages reported [16].

The rate of spine surgical procedures continues to increase worldwide [17]. It is known that patient success rates decline with each reoperation [1]. VersaWrap is a novel option unique from previous barrier (or nerve protector) technologies. Similar barrier (or nerve protector) technologies suggest easy-to-use applications and promising clinical outcomes but lack reports of conjunction use with medicinal agents [6]. When used in conjunction with medicinal agents, other technologies report reduced postoperative pain after spine procedures [10]. Yet, when used alone, these same technologies also report postoperative complications, such as disturbed muscle healing [9]. Surgeon experience in our representative case was that VersaWrap use allowed for minimal mechanical disruption and decreased the risk of complications during reoperation in the presence of scar tissue. These findings suggest that VersaWrap, when used with Depo-Medrol and Marcaine/epinephrine, was not associated with intraoperative or postoperative complications in this series.

The use of VersaWrap in surgical procedures is well-established [16,18-20]. Intraoperative local irrigation with medicinal agents during spine procedures is a common practice [14,15]. The irrigation activity will vary based on the procedure, patient characteristics, and combination of techniques and treatments. VersaWrap is often used in procedures where surgeons irrigate with anesthetics, antibiotics, or steroids, but the report of VersaWrap in conjunction with medicinal agents is unknown, to our knowledge, until now.

While the present series reports compatibility of VersaWrap with medicinal agents, there are some limitations that warrant consideration. First, this series provides only one representative case. Second, the synergistic impact that VersaWrap and medicinal agents have on one another still needs to be quantified. This series did not consider clinical evidence that the viscous biopolymers in VersaWrap provide a vehicle for localized delivery of medicinal agents that are currently only administered systemically. An additional study reviewing the impact that the viscous VersaWrap gel may have on sustained local release of medicinal agent is warranted. Despite these limitations, the present series allows for a second-look assessment of the initial VersaWrap application and provides surgeon experience that any scar tissue present was easy to remove without significant mechanical disruption.

## Conclusions

The integration of VersaWrap with medicinal agents represents a promising approach to manage scar tissue tethering of the nerve root to the surrounding tissues in spine procedures. The ability to apply medicinal agents alongside VersaWrap introduces a new sequela that optimizes personalized treatment options based on specific patient anatomy or conditions and reduces the need for systemic administration of drugs. We expect continued development of VersaWrap application techniques in innovative spine procedures, giving peer-to-peer insight and advancement of use to allow more patients the option of VersaWrap.
